# Lactic acid bacteria-specific induction of CD4^+^Foxp3^+^T cells ameliorates shrimp tropomyosin-induced allergic response in mice via suppression of mTOR signaling

**DOI:** 10.1038/s41598-017-02260-8

**Published:** 2017-05-16

**Authors:** Linglin Fu, Jixiang Peng, Shushu Zhao, Yan Zhang, Xiurong Su, Yanbo Wang

**Affiliations:** 1 Food Safety Key Laboratory of Zhejiang Province, School of Food Science and Biotechnology, Zhejiang Gongshang University, Hangzhou, 310018 P.R. China; 2 Laboratory of Mucosal Immunology and Food Research, School of Food Science and Biotechnology, Zhejiang Gongshang University, Hangzhou, 310018 P.R. China; 3 Hebei Food Inspection and Research Institute, Shijiazhuang, 050091 P. R. China; 4 School of Marine Sciences, Ningbo University, Ningbo, 315211 P.R. China

## Abstract

The beneficial effects of probiotics have been described in allergic sensitization and diseases; however, many questions remain unanswered, such as characteristics of the most effective strains in modulation of allergic responses and how orally administered probiotics affect the systemic immune system. In the present work, oral administration of five lactic acid bacteria strains showed variable effects on protection against the allergic reaction in a mouse model of food allergy to shrimp tropomyosin (ST). The most effective anti-allergic strain, *Bacillus coagulans* 09.712 (Bc), greatly improved epithelial barrier function and increased lymphocytes proliferation. Moreover, Bc suppresses ST sensitization by altering Th1/Th2/Treg balance as a result of strong induction of CD4^+^Foxp3^+^Tregs in combination with IL-10 producing. Bc-specific induction of CD4^+^Foxp3^+^ Tregs also suppresses Th17 pro-inflammatory response in this mouse model. Finally, the intake of Bc suppresses mTOR activation and thus the phosphorylation of downstream factors. Inhibition of mTOR signaling by Bc further results in FOXP3 up-regulation and GATA-3 down-regulation, which, in turn, facilitate to control Th2-predominant and Th17 pro-inflammatory responses caused by ST. Our work provides further characterization of the anti-allergic effects of probiotic LAB strains, and identifies new targets for preventive and curative treatment of food allergies.

## Introduction

Th2-regulated immune responses can be induced by food allergens, characterized by activation of mast cells or basophils and production of food protein-specific immunoglobulin E (IgE) resulting in the development of food hypersensitivity reactions^1^. IgE-mediated food allergy has been estimated to affect 1–2% of the adult population and up to 5–8% of children^2^ and has shown an increasing trend in several regions worldwide^3^. Among food allergies, seafood allergy represents a major concern as it is frequently associated with severe anaphylaxis and life-threatening reactions, with fish and shellfish being two of the “big eight” categories of food allergens^4^. Shrimp tropomyosin (ST) is the major heat-stable shrimp allergen and accounts for most of the allergenic activity of whole shrimp extract^5–7^.

Recently, experimental and clinical evidence supporting the efficacy of probiotic bacteria in the treatment of gastrointestinal disorders and allergic symptoms has triggered strong interest in the identification of novel strains and characterization of biological mechanisms behind the beneficial effects^8–13^. Probiotics, which could be introduced in natural host microbial communities, have a positive effect on health that contributes to nutritional physiology, modulation of inflammatory and hypersensitivity responses, or prevention of intestinal infections^14, 15^. However, results of clinical studies on the efficacy of prophylactic or therapeutic treatments with different bacterial strains in the context of allergic sensitization have been conflicting^16–18^, and the anti-allergic effects of probiotic bacteria are still not completely defined. Thus, many questions remain unanswered, such as which probiotic strains are the most effective in modulation of allergic responses and how orally administered probiotics affect the systemic immune system^19^.

In the original dogma of possible mechanism of their protective action, it was thought that probiotic bacteria can regulate the Th1/Th2 balance, and mainly induce Th1, thereby skewing the balance away from the Th2 cells that have an important role in allergic inflammation^20^. In recent years, the original role of probiotics in Th1/Th2 balance has now been expanded to include the induction and balance of additional Th cell subsets, such as Th9^21^, Th17^22^ and Foxp3^+^ regulatory T (Treg) cells^10, 23–25^. It has been proven that administration of a mixture of five probiotic strains mainly induces generation of CD4^+^Foxp3^+^ Tregs and increases the suppressor activity of naturally occurring CD4^+^CD25^+^ Tregs, which is directly mediated by regulatory dendritic cells (rDCs)^25^. However, it is unlikely that all probiotic bacteria are equally effective in inducing T regulatory cells *in vivo*.

Since it has been shown that Tregs have the ability to control and modify the development of allergic diseases by altering the sensitization and effector phases, great effort has been put into the identification of signaling pathways that modulate Treg induction. Based on MiRNome and transcriptome information, several signal transduction pathways, including T-cell receptor (TCR)-, Toll-like receptor (TLR)-, transforming growth factor-β (TGF-β)-, JAK/STAT (Janus kinase/signal transducers and activators of transcription)- and mammalian target of rapamycin (mTOR) signaling, might be involved in Treg regulation^26^. Notably, the mTOR signaling pathway has emerged as a critical regulator of immune function that integrates diverse micro-environmental signals to instruct T-cell differentiation^27^. Immunologically, mTORC1 activity leads to the increased activation of STAT4 and STAT3, and this in turn leads to increases in T-bet and RORγt in response to IL-12 and IL-6, respectively, which promote Th1 and Th17 differentiation. mTORC2 activity leads to the phosphorylation of Akt (at position S473) and SGK1, leading to their activation and in turn resulting in the phosphorylation and sequestration of FOXO proteins in the cytoplasm. This prevents the FOXO proteins from activating the transcription of target genes such as *foxp3*, a critical modulator of Treg development and function^26, 27^. Besides, mTORC2 activity also enhances STAT6 phosphorylation in response to IL-4 and subsequent GATA-3 expression and Th2 differentiation^27^. Nevertheless, the knowledge of cellular and molecular mechanisms underlying the preferential induction of Treg cell differentiation by probiotic strains involved in anti-allergic response is still incomplete.

In the preliminary study, we screened fermented milk curd or infant feces and isolated 21 potential probiotic lactic acid bacteria (LAB) strains with resistance to bile salts and extremes of acidity. Three strains of *Bacillus coagulans* 09.712, *Lactobacillus plantarum* 08.923 and *Bifidobacterium infantis* 11.322 displayed an *in vitro* capacity of inducing higher levels of IFN-γ and lower levels of IL-4 and IL-13 in macrophage-like THP-1 cells. The purpose of the present study was to examine the immunomodulatory capacity of oral administering these three strains in comparison to two commercial LAB strains (*Lactobacillus rhamnosus* CGMCC 1.3724 and *Streptococcus thermophilus* CGMCC 1.2471) on Th2 inflammation and anaphylaxis in a mouse model of food allergy to ST. Moreover, we sought to identify the potential molecular mechanism responsible for the preferential induction of CD4^+^Foxp3^+^ Tregs and improved balance of T cell subsets by the potent anti-allergic strain. Our results suggest that Treg cells and mTOR might be the useful targets in regulating food allergic responses.

## Results

### Different LAB strains exhibit individual protective effect against allergic reactions in ST-sensitized mice

**Figure 1:**
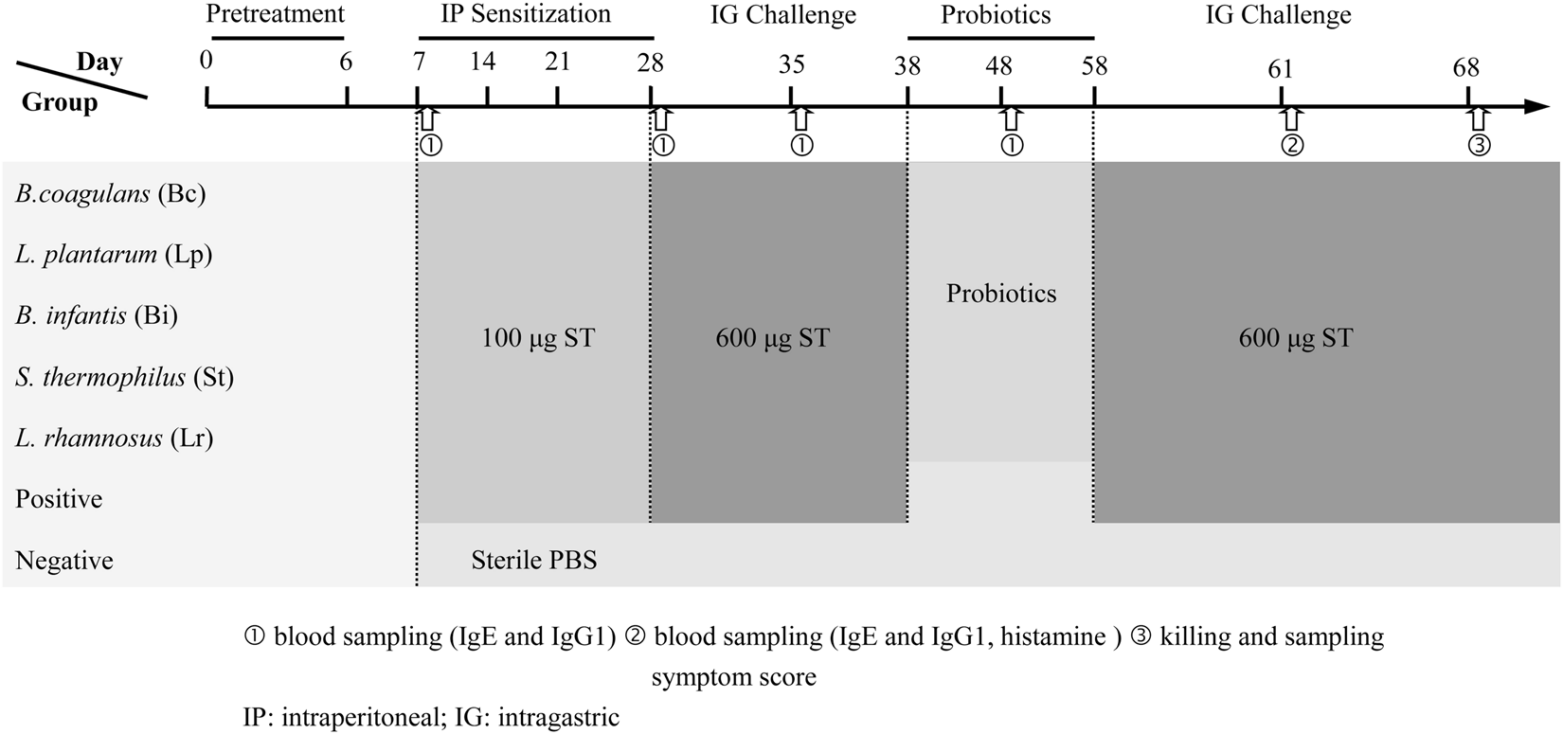
Experimental design of *in vivo* sensitization and probiotic treatment. Groups of mice (n = 8) were immunized by intraperitoneal (IP) injection of 100 μg purified ST/mouse on days 7, 14, 21 and 28, then challenged by receiving one intragastric gavage (IG) dose of ST (600 μg ST/mouse) on day 35. From day 38 to 58, sensitized mice were orally administered with different live probiotic bacteria daily. On day 61, mice were re-challenged by IG administration of ST. Blood samples were collected from the retro-orbital plexus on days 7, 28, 35, 48 and 61, and the appearance of symptoms of systemic anaphylaxis was observed on day 61. On day 68, mice were killed to collect blood, spleen, MLN and intestinal tissues. Negative control animals received equal amounts of sterile PBS at each sensitization and challenge point. Positive control received probiotic-free sterile PBS during bacteria treatment periods. In a separate experiment, three groups of mice (n = 8) including Bc-treated group, positive and negative controls were immunized following the same procedure as described above. Mice were sacrificed to collect spleen and intestinal tissues for immunoblotting, mTOR ELISA and immunohistochemical analysis.

**Figure 2:**
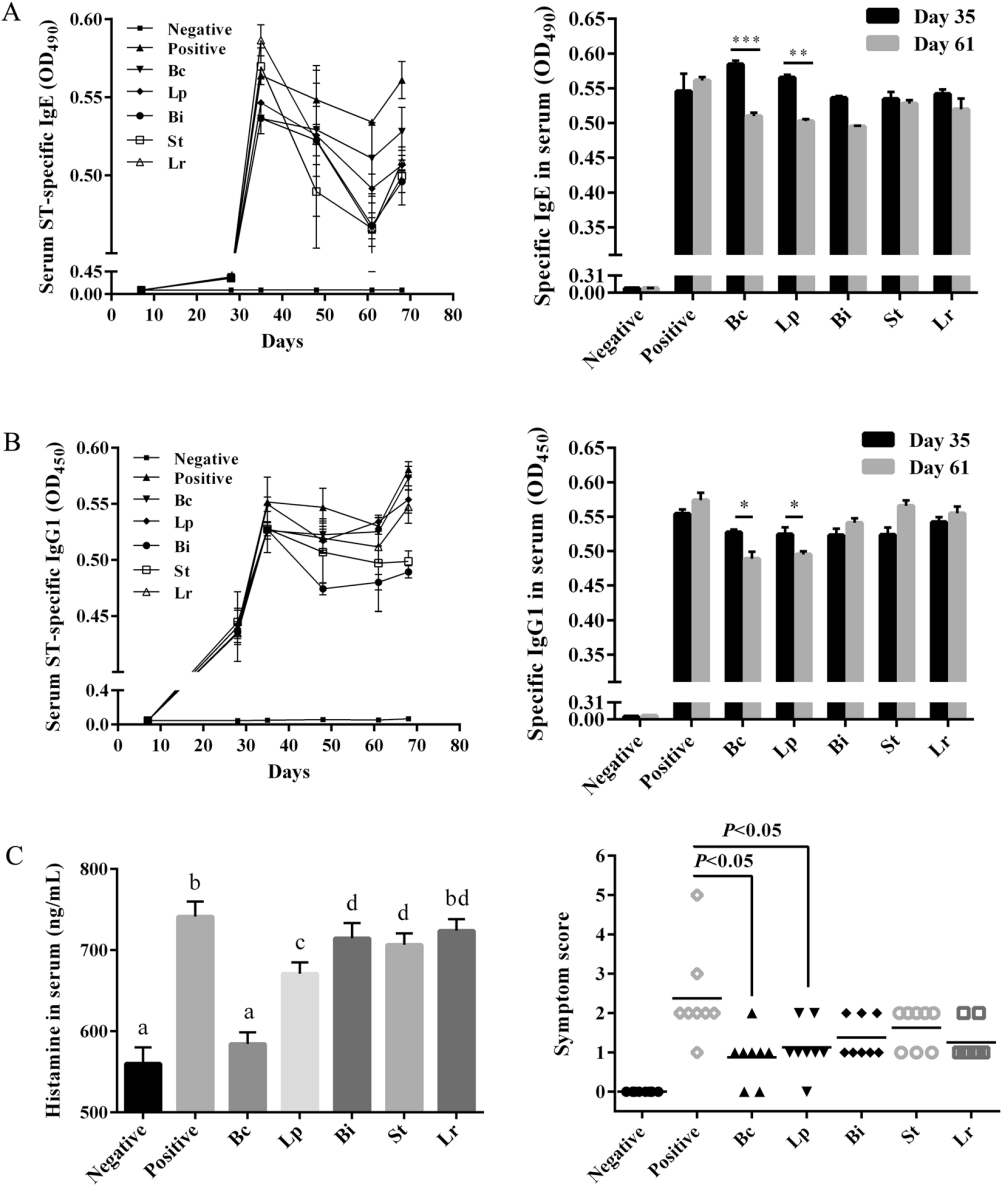
Variable effects of oral administration of five probiotic LAB strains on protection against ST-induced allergic reactions in mice. Time-course of ST-specific IgE (**A**) and IgG1 (**B**) antibodies in sera were determined by ELISA. (**C**) Histamine levels in sera collected at the second challenge on day 61were analyzed, and symptom scores were recorded during a 1-h observation period after challenge. The error bars indicate standard deviation (±SD). Statistical significance (*p* < 0.05) was marked by different letters in histamine level analysis. *p < 0.05, **p < 0.01, ***p < 0.001.

To develop a mouse model of shrimp tropomyosin hypersensitivity, BALB/c mice were sensitized and challenged with ST as shown in Fig. 1. We investigated whether these five different LAB strains were able to attenuate food anaphylaxis in this mouse model when administered by the oral route. ST-sensitized mice (positive control) produced high ST-specific IgE (sIgE) and IgG1 (sIgG1) levels since the first allergen challenge (day 35), as well as high levels of serum histamine (741.43 ± 18.44 ng/ml) and anaphylactic symptoms upon the secondary challenge (day 61) with severity ranging from 1 to 3 as measured by the scoring system (Fig. 2). The levels of sIgE and sIgG1 on day 61 were significantly lower (*p* < 0.05) in mice supplemented for 20 days with daily oral administration of Bc and Lp compared to the control, while no significant differences from the control were found in Bi-, Bt- and Lr-treated mice (Fig. 2A,B).

Different LAB strains also had individual capacities of suppressing serum histamine levels and anaphylactic symptoms caused by ST sensitization (Fig. 2C). In both Bc- and Lp-treated groups, serum histamine levels at the second challenge were significantly reduced (*p* < 0.01) compared to the control, and 5/8 mice had only slight cutaneous reaction with scratching and rubbing around the nose and head (score 1). However, these symptoms were obvious in Bi-, Bt- and Lr-treated groups with additional puffiness around the eyes and mouth (score 2). None of the mice from the negative control showed any symptoms of anaphylaxis during the course of sensitization and challenge.

### Anti-allergic probiotic LAB strains improve epithelial barrier functions after ST sensitization

**Figure 3:**
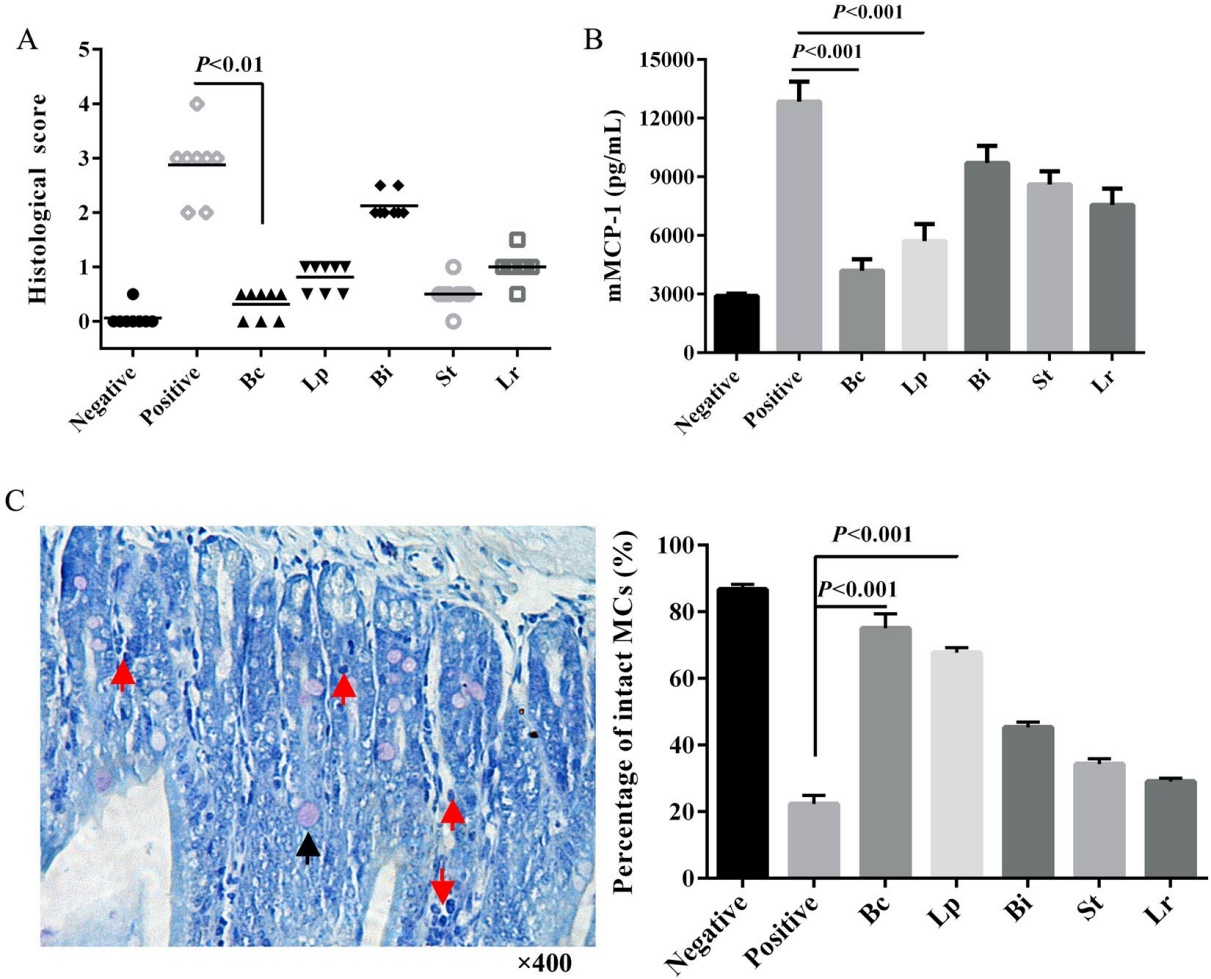
Histological and mast cell analyses of intestinal tissues after administration of different LAB strains during response to ST sensitization. (**A**) histological scores; (**B**) serum concentration of mouse mast cell protease-1 (mMCP-1); (**C**) representative intestinal specimens stained with toluidine blue (TB), and the percentage of intact mast cells (MCs) in lamina propria. Sections were photographed at ×400 magnifications using a light microscope. Intact and degranulated MCs are indicated by black and red arrows, respectively. The error bars indicate standard deviation (±SD).

Examination of intestine histology indicated that severe damage of epithelium occurred in the ST-sensitized mice based on disruption of epithelial architecture and sub-epithelial edema, flattened epithelium, reduced brush border, cuboidal epithelial morphology and accelerated shedding of cells from the epithelium (Fig. S2). Remarkably, treatment with Bc improved gut epithelium integrity under ST sensitization, with columnar morphology, thick epithelial folds and a well-developed brush border (Fig. S2), and histological score ranging from 0–1 (Fig. 3A). Different levels of epithelial damage were observed in other probiotic LAB strain-treated groups (Figs S2 and 3A). Moreover, serum concentrations of mouse mast cell protease-1 (mMCP-1) in Bc and Lp groups were significantly decreased (*p* < 0.001) compared to the control (Fig. 3B). Histological analysis of intestinal tissue showed many degranulated mast cells (MCs) in the ST-sensitized mice, and treatment with Bc and Lp alleviated degranulation of MCs (Fig. S3). The percentages of intact MCs in Bc- and Lp-treated mice were significantly (*p* < 0.001) higher than in the ST-sensitized group (Fig. 3C).

**Figure 4:**
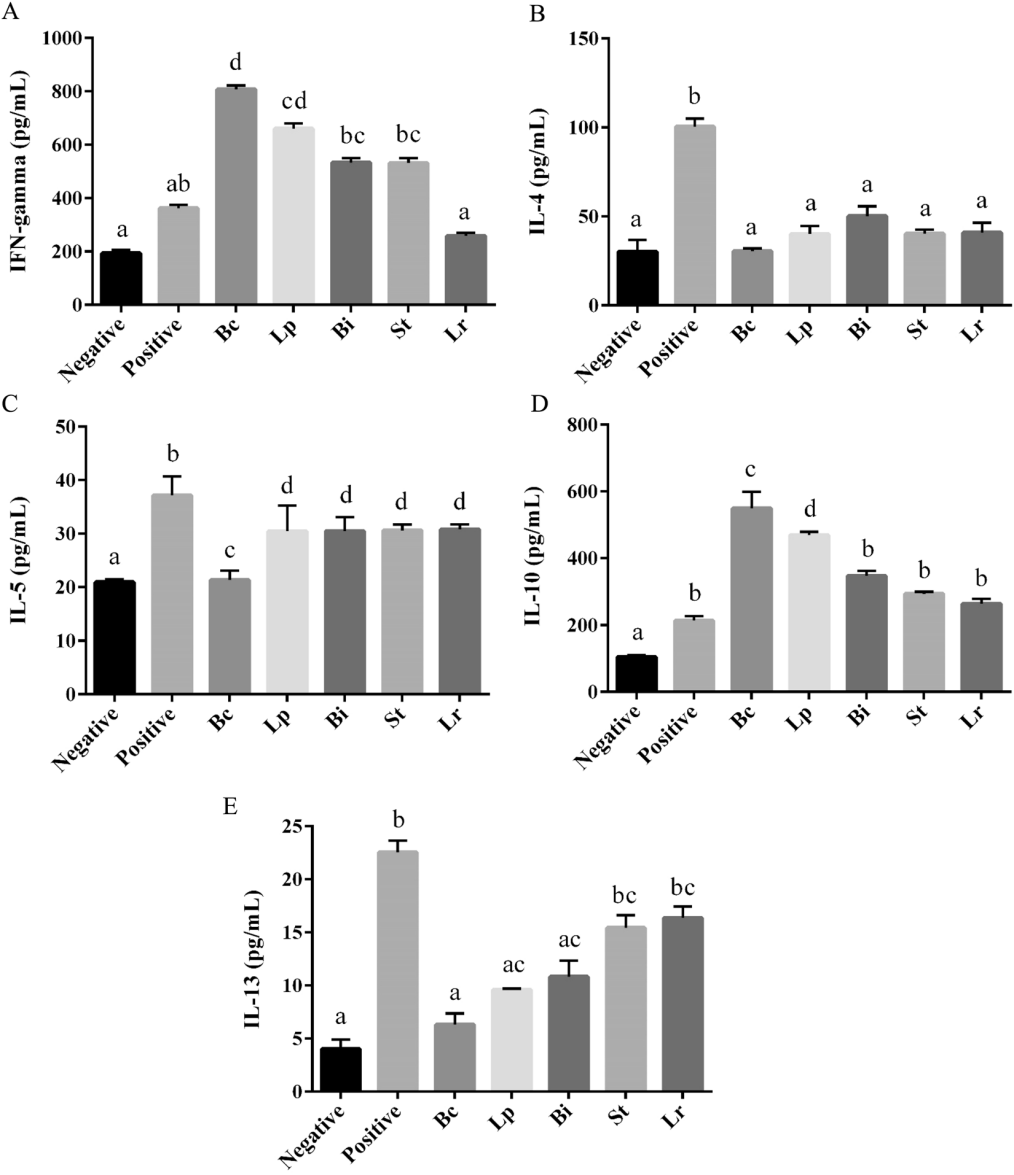
Influence of oral administration of probiotic LAB strains on cytokine production in MLN. After *in vitro* stimulation with ST, the levels of IFN-γ (**A**), IL-4 (**B**), IL-5 (**C**), IL-10 (**D**) and IL-13 (**E**) in the supernatants of MLN cells from the respective groups were measured. Results are expressed as means ± SD. Statistical significance (*p* < 0.05) was marked by different letters.

To further evaluate the effect of different LAB strains on the mucosal immune response to allergen ST, the levels of cytokines in the ST-stimulated MLN cell cultures were measured. IL-4, IL-5 and IL-13 production were significantly suppressed in the mice fed with any LAB strain used in comparison with the positive group (Fig. 4B,C,E). Levels of the cytokines IFN-γ and IL-10 were increased by LAB strains in the ST-sensitized mice (Fig. 4A,D). Notably, Bc displayed the strongest suppression of IL-4, IL-5 and IL-13 and greatest induction of IFN-γ and IL-10 (Fig. 4).

### Bc regulates the proliferation and function of lymphocytes after ST sensitization

Next, we tested whether the probiotic LAB strains affect the proliferation and effector function of T lymphocytes. Treatment with Bc and Lp effectively promoted the proliferation of total spleen lymphocytes (Fig. S4). In addition, administration of Bc reduced the levels of Th2 (IL-4, IL-5 and IL-13) cytokines, and significantly (*p* < 0.001) enhanced the levels of Th1 (IFN-γ) and Treg (IL-10) cytokines secreted from spleen T cells. Uniquely, splenic IL-17A production was greatly (*p* < 0.01) decreased in Bc group while no significant differences from the control were observed in other LAB strain-treated groups (Table 1).

**Table 1:** Influence of oral administration with probiotic LAB strains on cytokine production from spleen T cells.

Groups	Cytokines (pg/ml)					
	IFN-γ	IL-4	IL-5	IL-13	IL-10	IL-17A
Negative	582 ± 95	126 ± 18	21 ± 4	18 ± 6	159 ± 22	12 ± 2
Positive	1022 ± 117	223 ± 38	53 ± 9	39 ± 2	288 ± 17	36 ± 7
*B. coagulans* (Bc)	1360 ± 188***	158 ± 27***	32 ± 6*	25 ± 4**	720 ± 35***	14 ± 5**
*L. plantarum* (Lp)	1129 ± 102*	178 ± 12**	49 ± 8	37 ± 2	556 ± 28**	37 ± 2
*B. infantis* (Bi)	1261 ± 193**	215 ± 16	45 ± 9	29 ± 3*	291 ± 31	34 ± 3
*B. thermophilus* (St)	1109 ± 97*	217 ± 29	46 ± 7	38 ± 5	298 ± 16	36 ± 4
*L. rhamnosus* (Lr)	1044 ± 121	220 ± 15	46 ± 6	36 ± 6	290 ± 12	35 ± 3

Results were expressed as means ± SD, *p < 0.05, **p < 0.01 and ***p < 0.001 compared with the positive group.

### Bc increases CD4^+^Foxp3^+^ Tregs and suppresses Th2 response in ST-sensitized mice

**Figure 5:**
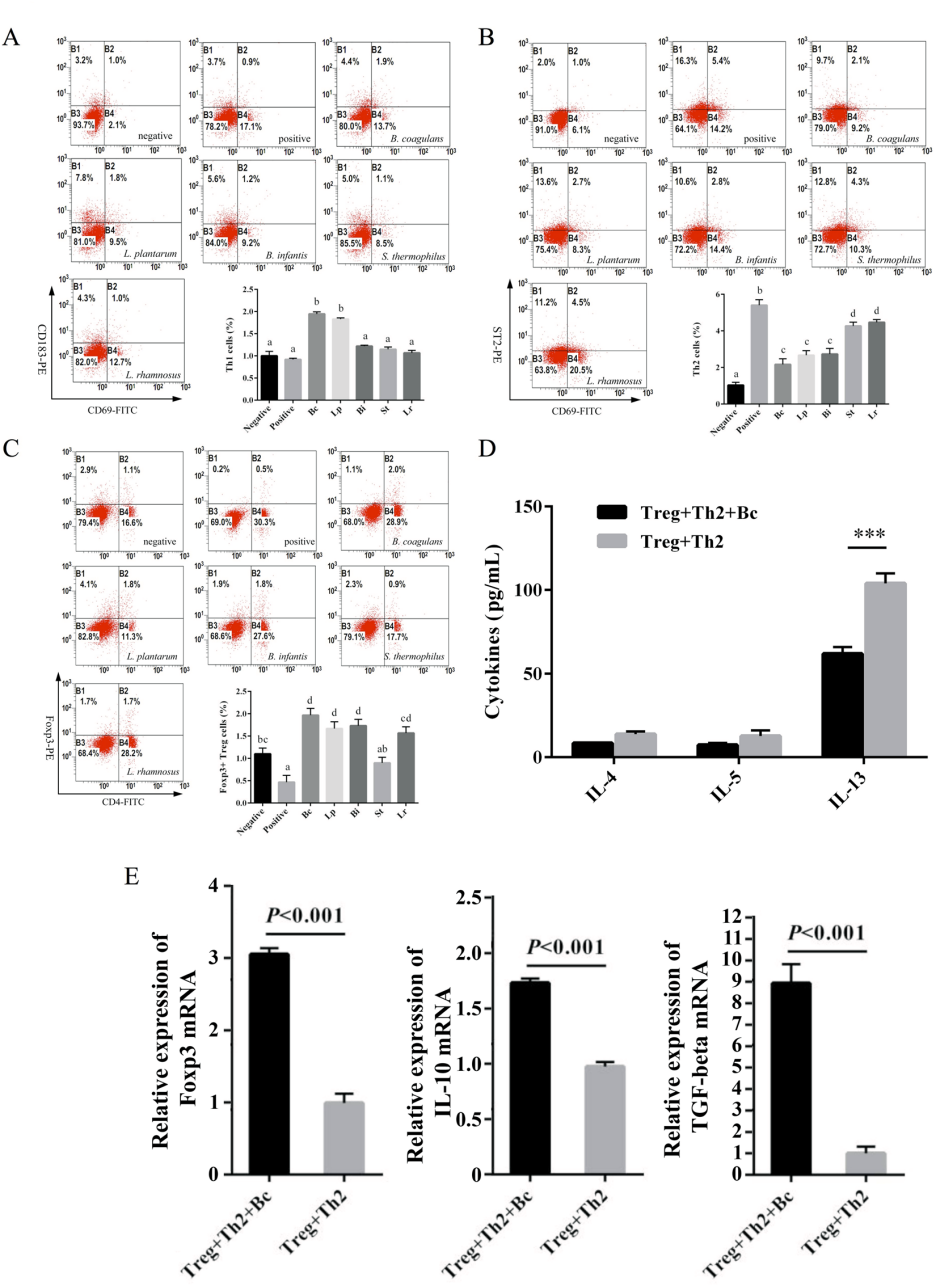
Administration of Bc increases CD4^+^Foxp3^+^ Tregs for modulating ST-induced T cell responses. The proportion of Th1 (**A**), Th2 (**B**) and Foxp3^+^ Treg (**C**) populations in spleen of the controls or probiotic-treated mice were analyzed by FACS. IL-4, IL-5 and IL-13 production (**D**) was evaluated by ELISA in culture supernatants. Levels of *Foxp3*, *TGF-β* and *IL-10* gene expression (**E**) were analyzed by real-time PCR. Relative expression was calculated using the 2^−ΔΔCt^ method after normalizing to the housekeeping transcript *hprt*. Results are expressed as means ± SD. Statistical significance (*p* < 0.05) was marked by different letters in T cell subset analysis. *p < 0.05, **p < 0.01, ***p < 0.001.

Administration of Bc significantly (*p* < 0.001) increased the population of CD4^+^Foxp3^+^ T cells in the spleen and reduced the percentage of splenic Th2 cells compared with positive control or other individual strain (Fig. 5B,C). The population of splenic Th1 cells was also enhanced in the Bc- and Lp-treated mice (Fig. 5A). In addition, treatment with Bc maintained the balance of Th1/Th2, Th1/Treg and Th2/Treg compared with controls (Fig. S5).

Next, we tested whether Bc-induced Tregs could suppress Th2 response under ST sensitization. Co-culture of Treg and Th2 cells sorted by FACS (Fig. S6) from naïve mouse spleen was stimulated with/without Bc followed by ST sensitization *in vitro* to measure the transcriptional and translational levels of different cytokines. Treatment with Bc resulted in a marked reduction in IL-4 and IL-13 secreting levels (Fig. 5D), while the *tgf-β*, *il-10* and *foxp3* mRNA expression were significantly (*p* < 0.001) upregulated with Bc stimulation compared to without Bc treatment (Fig. 5E).

### Bc-induced CD4^+^Foxp3^+^ Tregs control Th17 response in ST-sensitized mice

**Figure 6:**
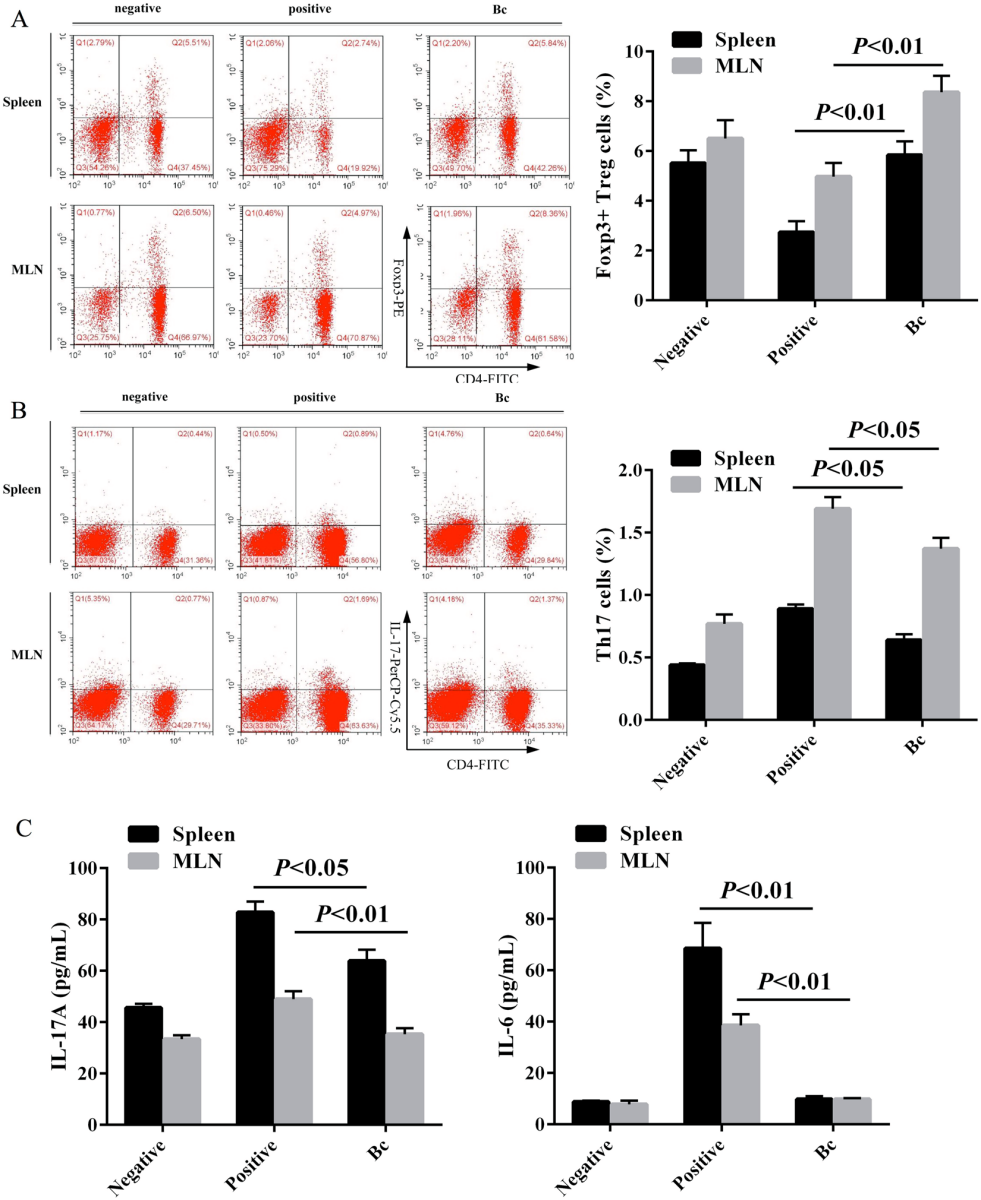
Bc-induced CD4^+^Foxp3^+^ Tregs control Th17 response in ST-sensitized mice. The proportion of Foxp3^+^ Treg (**A**) and Th17 (**B**) populations in both spleen and MLN of the controls or Bc-treated mice were analyzed by FACS. IL-17A and IL-6 production (**C**) were evaluated by ELISA in splenocytes and MLN cell culture supernatants. Results are expressed as means ± SD.

To corroborate the unique role of Bc in Th17 response following ST sensitization (Table 1), we also explored the effect of Bc administration on Th17 differentiation and Treg/Th17 balance in both spleen and MLN. Treatment with Bc promoted the induction of splenic and MLN-CD4^+^Foxp3^+^ Tregs (Fig. 6A), while the population of Th17 cell subset markedly (*p* < 0.05) declined in Bc group compared with positive control in both spleen and MLN (Fig. 6B). Therefore, analysis of the ratios of Treg and Th17 cell subsets showed that the intake of Bc maintained the balance of Treg/Th17 cells compared with controls (Fig. S7). The production of Th17 cytokines (IL-17A and IL-6) detected in both splenocyte and MLN cell cultures were dramatically suppressed following administration of Bc compared to the positive control (Fig. 6C).

### Bc administration inhibits phosphorylation of mTOR signaling molecules to facilitate Treg induction

**Figure 7:**
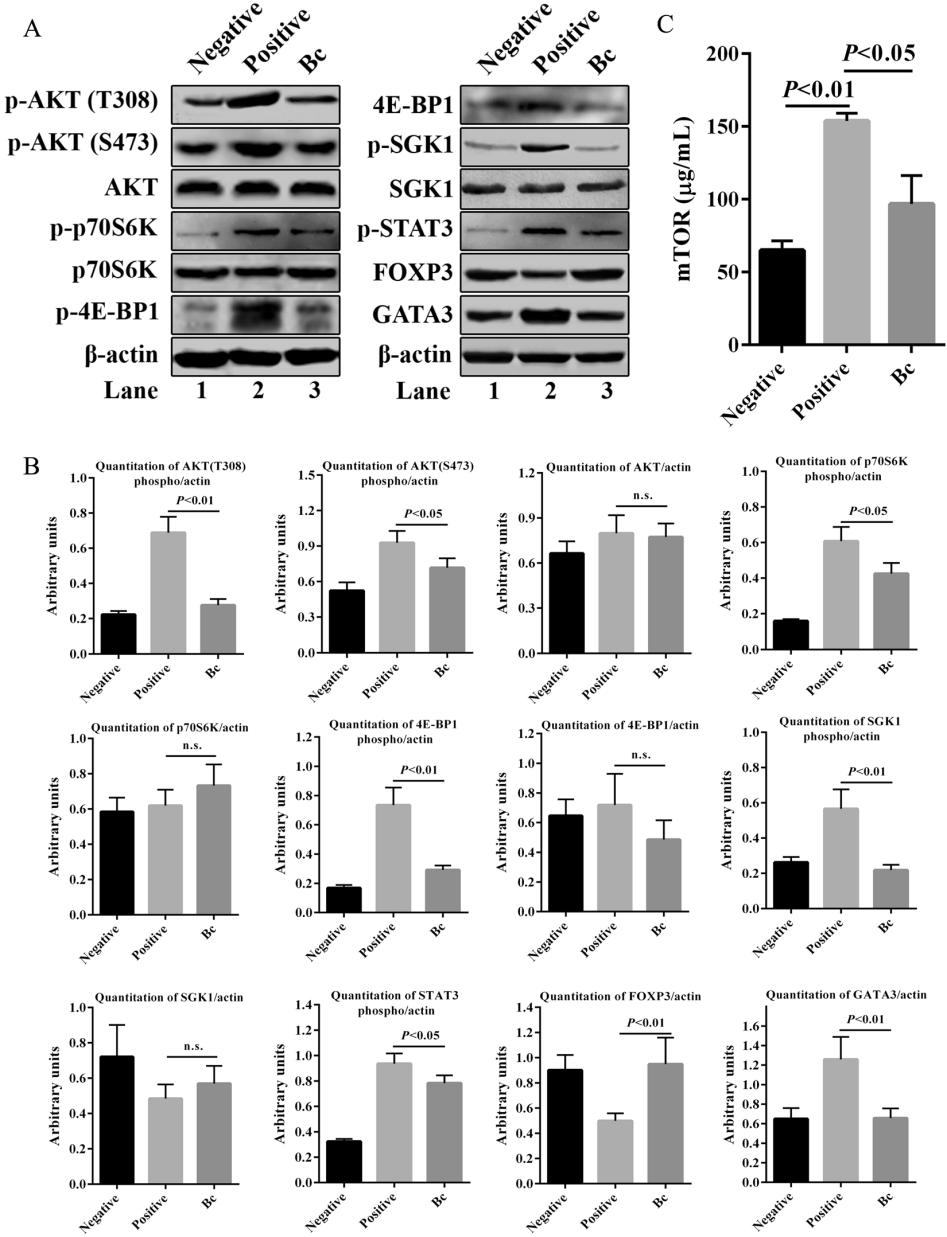
Treatment with Bc mediates mTOR signaling blockade leading to up-regulation of Foxp3 expression. (**A**) Splenocytes were lysed and immunoblotted with antibodies to SGK1^phospho Ser422^, pan-Akt^phospho T305/308/309^, p70S6K^phospho Thr389^, 4E-BP1^phospho Thr37/46^, Akt^phospho Ser473^, Stat3^phospho Tyr705^, Foxp3, p70S6K, 4E-BP1, Akt, SGK1, GATA3 and **β**-actin. (**B**) Quantitation of band intensities are shown, respectively. (**C**) The levels of mTOR were analyzed by ELISA. The results shown are representative of more than three experiments with similar results. Data are expressed as means ± SD. n. s., no significance. Full-length blots are presented in Supplementary Fig. S9.

To further uncover the potential regulators of Bc-specific induction of Tregs and other T cell subset differentiation, we investigated mTOR activity and the expression levels of mTOR signaling-related molecules regulated by Bc administration upon ST challenge *in vitro*. Phosphorylation of pan-Akt^T305/308/309^, Akt^Ser473^, p70S6K^Thr389^, 4E-BP1^Thr37/46^ and SGK1^Ser422^ were significantly suppressed by the intake of Bc upon ST sensitization in mice (Fig. 7A,B). However, no differences were observed in the expression levels of non-phosphorylated AKT, p70S6K, 4E-BP1 and SGK1 molecules among the experimental groups. Meanwhile, the activity of mTOR remarkably (*p* < 0.05) declined in Bc group compared to positive control (Fig. 7C). In addition, the expression of phosphorylated STAT3^Tyr705^ and Th2 lineage-specific transcription factor GATA3 were down-regulated, while FOXP3 was up-regulated in Bc administered mice (Fig. 7A,B).

Next, to demonstrate the effect of Bc on inhibition of STAT3^phosphoTyr705^ and GATA3 *in vivo*, lamina propria was immunostained. A significantly lower expression of STAT3^phosphoTyr705^ and GATA3 was observed in Bc-administered mice compared with positive control (Fig. S8).

## Discussion

There is a significant difference in clinical effects with long-term supplementation of probiotics in relation to species and even the strain of LAB. To date, LAB administration has been shown to be an effective strategy for the prevention of allergic sensitization mostly in mice, but these models have been useful for identifying the best strains^28^. Besides, variable effects of LAB in the prevention and treatment of allergic diseases also show great differences in mechanisms of their action^19^. Therefore, in this study, we demonstrated differential effects in relation to molecular action of oral administration of five individual LAB strains in alleviating Th2-driven intestinal inflammation and other symptoms associated with food anaphylaxis in a murine model induced by a major shrimp allergen ST. Indeed, in preliminary *in vitro* experiment, Bc, Lp and Bi were able to down-regulate Th2-biased responses. Nevertheless, the present study showed that oral supplementation with Bc and Lp significantly ameliorates anaphylaxis symptoms while no obvious protective effect of Bi was found *in vivo*. The consistency of *in vitro* results with *in vivo* behavior of Bi strain remains to be clarified.

Possible mechanisms of probiotic protective action include: (i) the enhancement of the epithelial barrier function^29^, and (ii) the induction of regulatory dendritic cells (rDC), which, in turn, promotes generation of Foxp3^+^ Tregs^25^. Firstly, epithelium is a crucial regulator of intestinal immune homeostasis. The epithelial integrity and mucosal immune cells located beneath the epithelium play critical roles in regulating the mucosal barrier. Most of the antigen might be sampled at the surface of the villus epithelium by DCs, and traffic to the MLN and then prime T-cell responses^29^. Innate lymphoid cells and mast cells (MCs), in particular, orchestrate the mucosal regulatory system to create a mutually beneficial environment for both the host and the microbiota^30^. Barrier dysfunction drives Th2 responses in food anaphylaxis, atopic disorders or eosinophilic esophagitis^31^. Our findings showed that LAB strains (Bc and Lp) with notable anti-allergic effect against ST sensitization greatly restored the intestinal epithelial barrier functions by improvement of gut epithelial integrity, suppression of MCs degranulation in lamina propria, and skewing of the immune response from a Th2 to a Th1 polarization in MLN. Secondly, we found that treatment with the best anti-allergic strain (Bc) could regulate systemic immune responses in an allergic environment by promoting the proliferation of splenic lymphocytes, balancing Th1/Th2/Treg cytokines, and suppressing pro-inflammatory cytokine (IL-17A and IL-6) secretion.

Furthermore, we present evidence that ingestion of Bc can greatly generate CD4^+^Foxp3^+^ Treg population, which is consistent with the results from different probiotic strains described in other studies^10, 25, 32^. Treg cells have a key role in promoting and maintaining tolerance to allergens by regulating both innate and adaptive allergen-triggered immune responses^33^. It is worth noting that strains of Bi, Bt and Lr incapable of reducing allergic symptoms in an effective way can also increase the number of Tregs by administration in mice. We found that the immunomodulatory action of Bc was achieved by increasing the expression of Th1 (IFN-γ) and Treg (IL-10) cytokines, and suppressing the expression of Th2 (IL-4, IL-5 and IL-13) cytokines in T cells. However, it can be noticed that the levels of IL-10 in both spleen and MLN showed no significant differences in Bi-, Bt- and Lr-treated mice compared to positive control. Collectively, probiotic LAB suppress ST sensitization by improving Th1/Th2/Treg balance and inhibiting Th2-predominant immunologic reactivity. This occurs only when, in combination with IL-10 production, probiotic LAB induce Foxp3^+^ Tregs. Recent evidence also mentioned the role of Treg-mediated control of Th17 response in intestinal immune homeostasis^34^. Th17 cells play a significant role in the development of chronic inflammatory diseases, including allergic and autoimmune diseases. Our findings show that administration of Bc controls ST-mediated Th17 cell over-expansion by induction of Tregs, thus reducing pro-inflammatory cytokine production and improving Th17/Treg balance. It has been shown that Th17 cells enhance not only neutrophilic airway inflammation but also Th2 cell-mediated eosinophilic airway inflammation in a mouse model of asthma^35^. Moreover, it is also found that oral administration of *Lactobacillus gasseri* can attenuate major characteristics of allergen-induced airway inflammation by suppressing IL-17 immune response in a mouse model of allergic asthma^36^. Therefore, Bc-specific induction of CD4^+^Foxp3^+^ Tregs not only inhibits Th2 inflammation and anaphylaxis, but also suppresses Th17 pro-inflammatory responses in this mouse model of food allergy to ST.

**Figure 8:**
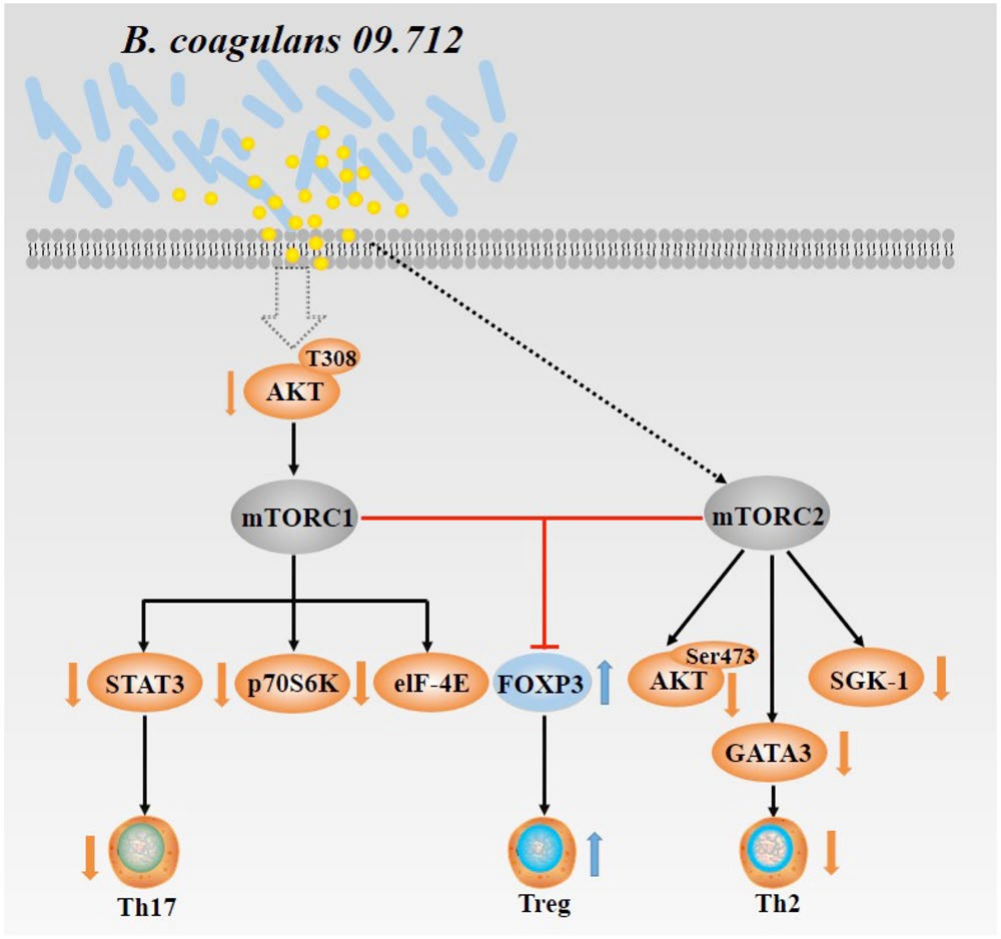
A schematic diagram of Bc-specific induction of regulatory T cells via suppression of mTOR signaling pathway. The intake of Bc probably suppresses mTOR (mTOR1 and mTOR2) activation that leads to decreased phosphorylation of p70S6K, 4E-BP1, STAT3, AKT (Ser473) and SGK1, as well as up-regulation of transcription factor FOXP3 and down-regulation of transcription factor GATA-3. These events, in turn, facilitate the induction of CD4^+^Foxp3^+^ Tregs and control of Th2-predominant and Th17 pro-inflammatory responses caused by ST.

Little information is available on the mechanism by which administration of probiotics generates regulatory T-cell populations. Indeed, administration of probiotics induced rDCs, which, in turn, promoted generation of CD4^+^Foxp3^+^ T cells^25^. But what signaling molecules would be involved in promoting the induction of Tregs by probiotics? mTOR with two distinct complexes, mTORC1 and mTORC2, is now appreciated to be a central regulator of diverse immune cells by promoting differentiation, activation, and function in T cells, B cells, and antigen-presenting cells^27^. Because mTOR inhibition has been reported to promote CD4^+^Foxp3^+^ Tregs^37^, this specific anti-allergic strain (Bc) may influence mTOR signaling to regulate Treg expansion during sensitization. To verify this hypothesis, we examined the effect of Bc administration on the phosphorylation of key factors involved in the mTOR signaling pathway. On the basis of our observations, we propose that the intake of Bc probably suppresses mTOR (mTOR1 and mTOR2) activation, leading to decreased phosphorylation of p70S6K, 4E-BP1, STAT3, AKT (Ser473) and SGK1, as well as up-regulation of transcription factor FOXP3 and down-regulation of transcription factor GATA-3. These events facilitate the induction of CD4^+^Foxp3^+^ Tregs and control Th2-predominant and Th17 pro-inflammatory responses caused by ST (Fig. 8).

While the exact mechanism responsible for the variable effects of different probiotic strains in the suppression of allergic responses is not well understood, our current study has shown that effective immunotherapeutic probiotic LAB may exert their influence via a modulation of regulatory T cells in combination with enhancement of IL-10 production. Administration of a unique strain Bc can substantially potentiate epithelial barrier function, and then stimulate the generation of Foxp3^+^ Tregs as well as the production of the anti-inflammatory cytokine IL-10, thereby leading to the reduction of food allergic inflammation and symptoms caused by ST. We also demonstrated, for the first time in this system, that induction/maintenance of bacterial-specific Tregs could be regulated by mTOR signaling blockade to facilitate ST desensitization. Our work has identified novel probiotic LAB strains in ST-sensitized mouse model and characterized the mechanism of probiotic effects in the prevention and treatment of food allergies.

## Materials and Methods

### Ethics statement

This study was carried out in strict accordance with the recommendations in the National Guide for the Care and Use of Laboratory Animals of China. All animal procedures were approved by the Institutional Animal Care and Use Committee of Zhejiang Provincial Experimental Animal Public Service System.

### Shrimp tropomyosin

Tropomyosin was extracted and purified from the shrimp *Penaeus monodon*. Whole bodies of peeled frozen shrimps were minced in a stainless steel grinder followed by preparation of dried acetone powder. Acetone powder was extracted overnight with 1 M KCl and 0.5 mM dithiothreitol (DTT), pH 7, and centrifuged at 5000 *g* for 15 min. The supernatant was adjusted to pH 4.6 and stirred for 30 min. After centrifugation, the precipitate was extracted one more time for 2 h and centrifuged. The pooled supernatants were cooled to 4 °C, adjusted to pH 4.6 and stirred for 30 min. After centrifugation at the above conditions, the precipitate was dissolved in extraction buffer, adjusted to pH 7 and stirred for 20 min at room temperature. After removing insoluble material by centrifugation, the isoelectric precipitation at pH 4.6 and dissolution of the precipitate in extraction buffer at pH 7 were repeated twice. The final precipitate was dissolved in 0.5 mM DTT, pH 7, and dialyzed against PBS, pH 7.2. Further purification was obtained after precipitation with 55% ammonium sulphate saturation. The precipitate was dissolved in 0.5 mM DTT, dialyzed against 2 mM 2-mercaptoethanol (2-ME) and lyophilized. The concentration of ST protein was determined by the BCA assay (Pierce, Rockford, USA) with bovine serum albumin (BSA) as the standard. The purity of purified ST protein was analyzed by SDS-PAGE (Fig. S1).

### Bacterial strains and culture conditions

The potential probiotic LAB strains of *B. coagulans* 09.712 (Bc) and *L. plantarum* 08.923 (Lp) were originally obtained from fermented milk curd, and *B. infantis* 11.322 (Bi) from infant fecal samples, and kept in our own lab. The commercial strains of *L. rhamnosus* CGMCC 1.3724 (Lr) and *S. thermophilus* CGMCC 1.2471 (St) were purchased from China General Microbiological Culture Collection (CGMCC).

Bc was cultivated in Nutrient Broth (NB) medium at 37 °C for 48 h. All other probiotic strains were routinely grown in de Man, Rogosa, Sharpe (MRS) broth (Oxoid, Basingstoke, UK) at 37 °C for 48 h. To test the effect of single strains, each strain was administered to mice at a concentration of 1 × 10^9^ CFU per 100 µl.

### Animals

Six-week-old female BALB/c mice (SPF) were purchased and housed in the Animal Care Unit of Hangzhou Normal University. Mice were kept at a constant temperature of 23 ± 1 °C, relative humidity of 55 ± 5% and under a regular cycle (light/dark = 12 h/12 h). The sterile food and water were given ad libitum. Fifty-six mice were divided randomly into seven groups, and mice in each group were assigned to two cages (4 mice per cage), being kept on a tropomyosin-free diet during the course of the study. All mice were housed in the standard cages for one week before the experiments began.

### *In vivo* experimental protocols

Groups of mice (n = 8) were immunized by intraperitoneal (IP) injection of 100 μg purified ST/mouse together with incomplete Freund’s adjuvant (Sigma-Aldrich, St. Louis, USA) in a total of 500 μl of sterile phosphate-buffered saline (PBS) on days 7, 14, 21 and 28 (Fig. 1). One week after the last immunization (day 35), mice were challenged by receiving one intragastric gavage (IG) dose of ST (600 μg ST/mouse). Negative control animals received equal amounts of sterile PBS with Freund’s adjuvant at each sensitization and challenge point. From day 38 to 58, sensitized mice were orally administered with different live probiotic bacteria (10^9^ CFU, 100 µl per mouse) daily (Fig. 1). Negative and positive control groups received 100 μl/mouse of probiotic-free sterile PBS. On day 61, mice were re-challenged by IG administration of ST as described above.

Mice were bled from the retro-orbital plexus on days 7, 28, 35, 48 and 61. Serum samples were individually collected and stored at −20 °C until analysis. The appearance of symptoms of systemic anaphylaxis was observed within 1 h after re-challenge on day 61. On day 68, mice were killed to collect blood, spleen, mesenteric lymph node (MLN), duodenum, jejunum and ileum.

A separate experiment including three groups of mice (n = 8) that were designated as positive control, negative control and *B. coagulans* (Bc) group was performed using the same procedure as described above. Subsequently, mice were sacrificed to collect spleen and intestinal tissues. After *in vitro* culture and stimulation of splenocytes, cell lysates were harvested for the subsequent immunoblotting and mTOR ELISA analysis. Intestinal tissues were used for immunohistochemistry staining.

### Analysis of immunoglobulins and cytokines

Individual serum samples or cell culture supernatants were assayed for ST-specific IgE and IgG1 antibody response as described^38^. Plates were coated with ST (5 μg/ml) in carbonate/bicarbonate buffer (pH 9.6) overnight at 4 °C. The secondary antibodies used in the ELISA tests were rat anti-mouse IgE-HRP (1:8000, SouthernBiotech) and rat anti-mouse IgG1-HRP (1:8000, SouthernBiotech).

The levels of IFN-γ, IL-4, IL-5, IL-10, IL-13, IL-6 and IL-17A in the cell culture supernatants were measured by ELISA kits according to manufacturers’ instructions (eBioscience, San Diego, USA). The mTOR activity in the cell lysates were analyzed by mTOR (pSer2448) ELISA kit (Abcam).

### Measurement of histamine levels in sera

Histamine was assayed on post-challenge sera using an enzyme immunoassay kit (Baomanbio, Shanghai, China) according to manufacturer’s instructions. Samples were analyzed at 1:200 dilution.

### Measurement of mouse mast cell protease-1 levels in sera

Serum concentration of mouse mast cell protease-1 was measured by Mouse MCPT-1 (mMCP-1) ELISA Ready-SET-Go enzyme immunoassay kit (eBioscience, San Diego, USA) according to manufacturer’s instructions. Samples were analyzed at 1:20 dilution.

### Evaluation of symptoms

Symptoms were evaluated in the range 0–5 according to the scoring system as described previously^39^: 0, no symptoms; 1, scratching and rubbing around the nose and head; 2, puffiness around the eyes and mouth, diarrhoea, pilar erection, reduced activity and/or decreased activity with increased respiratory rate; 3, wheezing, laboured respiration and cyanosis around the mouth and the tail; 4, no activity after prodding or tremor and convulsion; 5, death.

### Histological analysis

At necropsy, the duodenum, jejunum, and ileum were excised and gently flushed with PBS. Then, the samples were fixed in neutral-buffered 10% formaldehyde and embedded in paraffin. Five-micrometer sections were stained with hematoxylin and eosin for tissue morphology or toluidine blue for degranulated mast cell.

The specimens were examined histologically for scoring the degree of intestinal injury based on a “0–4” scale: 0, no histological damage; 1 (mild), slight submucosal and/or lamina propria separation; 2 (moderate), moderate separation of the submucosa and/or lamina propria and/or edema in the submucosa and muscular layers; 3 (severe), severe separation of the submucosa and/or lamina propria and/or severe edema in the submucosa and muscular layers with regional villous sloughing; and 4 (necrosis), loss of villi and necrosis. The histological scoring was done blinded by two observers.

A degranulated mast cell was defined as a toluidine-positive cell with five or more distinct stained granules completely outside of the cell. The percentage of intact mast cells was calculated as: (number of total mast cells-number of degranulated mast cells)/number of total mast cells.

### Preparation of lymphocytes and *in vitro* stimulation

The spleen and MLN were mechanically disrupted into single-cell suspensions. After erythrocyte lysis, spleen lymphocytes were re-suspended in RPMI 1640 medium containing 10% FBS supplemented with 1% penicillin/streptomycin and 1% glutamine (Hyclone, Logan, Utah, USA). After that, 1 × 10^5^ cells (200 µl) were added and incubated in 96-well plates (Corning, NY, USA) with ST (10 µg/well) in triplicate for 48 h at 37 °C, and subsequently subjected to proliferation assay or cytokines analysis. Before the evaluation of cytokine production, MLN single-cell suspensions (2 × 10^6^ cells per 200 µl) were stimulated with ST (10 µg/well) for 48 h at 37 °C as well.

### Proliferation assay

The ST-stimulated spleen lymphocytes were incubated with 50 μl of microculture tetrazolium (2 mg/ml, Solarbio Science & Technology Co., Ltd, Beijing, China) for 4 h. The supernatants were then removed by centrifugation at 1000 g for 5 min. After incubation with 150 μl of dimethyl sulfoxide (DMSO, Solarbio Science & Technology Co., Ltd, Beijing, China) in a dark place for 15 min at room temperature, the absorbance was determined by an ELISA reader (Molecular Devices Versamax, Sunnyvale, CA, USA) at 578 nm. Results were expressed as optical densities at 578 nm (OD_578_).

### Flow cytometry

The following monoclonal antibodies were conjugated with fluorescein isothiocyanate (FITC) or phycoerythrin (PE) (eBioscience) and used to label cells for T cell subset analysis: anti-mouse CD4, CD69, CD183 (CXCR3), ST2 (IL-33R), CD25 (PC61). For intracellular staining of Foxp3, lymphocytes were pre-incubated with FcγR (CD16/CD32)-blocking monoclonal antibody (eBioscience) before staining for surface antigens. The cells were then fixed, permeabilized and stained with monoclonal antibodies using the Foxp3 staining set (eBioscience) according to the manufacturer’s instructions. For intracellular IL-17A (PerCP) staining, lymphocytes were cultured for 3 days in complete medium (RPMI 1640 containing 10% FCS, 100 U/ml penicillin, 0.1 mg/ml streptomycin) supplemented with 0.03 µg PMA/lonomycin. The lymphocytes were then stained with monoclonal antibodies against CD4, followed by intracellular staining for IL-17A using a Cytofix/Cytoperm kit (BD Bioscience). Cellular phenotypes were measured using MoFLo Astrios EQ (Beckman Coulter, Brea, CA, USA) flow cytometer with Summit 6.2.2 software.

Spleen cells were collected from naïve female BALB/c mice, and subjected to cell sorting with MoFLo Astrios EQ to isolate Treg and Th2 using monoclonal antibodies of FITC-labeled anti-mouse CD4, PE-labeled anti-mouse CD25, FITC-labeled anti-mouse CD69 and PE-labeled anti-mouse ST2 (IL-33R) (eBioscience).

### *In vitro* culture

Co-culture of Treg and Th2 (5 × 10^5^ cells/ml) was supplemented with/without Bc (10^6^ CFU/ml) for 36 h, then stimulated with ST (50 μg/ml) for another 48 h. The cell supernatant was collected for measurement of secreting cytokines of IL-4, IL-5 and IL-13, while cell lysates were extracted for mRNA and quantitative PCR of *Foxp3*, *TGF-β* and *IL-10* expression.

### Quantitative PCR

mRNA was isolated from lysed cells and cDNA prepared using the iScript cDNA synthese kit (BioRad, Hercules, USA). Quantitative PCR reaction was performed on a LightCycler® Nano thermal cycler (Roche Applied Science, Penzberg, Germany), using SYBR Green I kit or SYBR Green FastStart kit (Roche). The sequences of the primers used are presented in Table 2. Relative expression was calculated using the 2^−ΔΔCt^ method after normalizing to the housekeeping transcript *hprt*.

**Table 2:** List of primers used for real-time PCR analyses.

Genes	Sequence (5′-3′)
Mouse-*il-10*	F: GGTTGCCAAGCCTTATCGGA
	R: ACCTGCTCCACTGCCTTGCT
Mouse-*tgf-β*	F: ACCGCAACAACGCCATCTAT
	R: GCACTGCTTCCCGAATGTCT
Mouse-*foxp3*	F: AGAGTTCTTCCACAACATGGACTACTT
	R: GATGGCCCATGGATAAGG
Mouse-*hprt*	F: CTGGTGAAAAGGACCTCTCG
	R: TGAAGTACTCATTATAGTCAAGGGCA

### Immunoblotting

Spleen tissues from positive, negative and Bc groups were mechanically disrupted into single-cell suspensions. After erythrocyte lysis, spleen lymphocytes were re-suspended in RPMI 1640 medium containing 10% FCS supplemented with 100 U/ml penicillin and 0.1 mg/ml streptomycin. Then, splenocytes (5 × 10^5^ cell/ml) were incubated in 96-well plates with ST (10 µg/well) in triplicate for 48 h at 37 °C. Cell lysates were harvested by adding 100 µl of RIPA lysis buffer containing protease and phosphatase inhibitors (Thermo Fisher Scientific, Waltham, MA, USA) per well and incubating on a shaking platform for 10 min at 4 °C. After centrifuged at 10,000 rpm for 10 min at 4 °C, the supernatants were collected and estimated for protein concentration. After gel electrophoresis and transfer to nitrocellulose membranes, the lysates were blotted with primary antibodies to the phosphorylated forms of SGK1 (rabbit polyclonal anti-SGK1^phospho Ser422^, Abcam), Akt (rabbit polyclonal anti-pan-Akt^phospho T305/308/309^, Abcam), p70S6K (rabbit polyclonal anti-p70S6K^phospho Thr389^, Cell Signaling Technology), 4E-BP1 (rabbit polyclonal anti-4E-BP1^phospho Thr37/46^, Cell Signaling Technology), Akt (rabbit polyclonal anti-Akt^phospho Ser473^, Cell Signaling Technology) and Stat3 (rabbit polyclonal anti-Stat3^phospho Tyr705^, Cell Signaling Technology), as well as rabbit polyclonal anti-mouse Foxp3 (Abcam), p70S6K (Cell Signaling Technology), 4E-BP1 (Cell Signaling Technology), Akt (Cell Signaling Technology), SGK1 (Abcam), GATA3 (Abcam) and **β**-actin (Sigma-Aldrich). The blots were developed with fluorochrome-conjugated secondary antibodies and visualized using the Odyssey infrared fluorescence imaging system (LI-COR Biosciences, Lincoln, NE, USA). Band fluorescence intensities were quantified after background subtraction and used to calculate the changes in relative amounts of the corresponding proteins.

### Statistical analysis

Statistical significance of the data was determined by one-way analysis of variance using SPSS.16.0 (IBM SPSS, Armonk, NY, USA). A *p* value of < 0.05 was considered to be statistically significant.

## Electronic supplementary material

Lactic acid bacteria-specific induction of CD4+Foxp3+T cells ameliorates shrimp tropomyosin-induced allergic response in mice via suppression of mTOR signaling

## References

[1] Sokol CL, Barton GM, Farr AG, Medzhitov R (2008). A mechanism for the initiation of allergen-induced T helper type 2 responses. Nat. Immunol..

[2] Mills EN, Breiteneder H (2005). Food allergy and its relevance to industrial food proteins. Biotechnol. Adv..

[3] Burks AW (2012). ICON: food allergy. J. Allergy Clin. Immunol..

[4] Hajeb P, Selamat J (2012). A contemporary review of seafood allergy. Clin. Rev. Allergy Immunol..

[5] Daul CB, Slattery M, Reese G, Lehrer SB (1994). Identification of the major brown shrimp (*Penaeus aztecus*) allergen as the muscle protein tropomyosin. Int. Arch. Allergy Immunol..

[6] Leung PS (1994). Cloning, expression, and primary structure of *Metapenaeus ensis* tropomyosin, the major heat-stable shrimp allergen. J. Allergy Clin. Immunol..

[7] Ayuso R, Lehrer SB, Reese G (2002). Identification of continuous, allergenic regions of the major shrimp allergen Pen a 1 (tropomyosin). Int. Arch. Allergy Immunol..

[8] Kalliomäki M (2001). Probiotics in primary prevention of atopic disease: a randomised placebo-controlled trial. Lancet.

[9] Wohlgemuth S, Loh G, Blaut M (2010). Recent developments and perspectives in the investigation of probiotic effects. Int. J. Med. Microbiol..

[10] Schiavi E (2011). Oral therapeutic administration of a probiotic mixture suppresses established Th2 responses and systemic anaphylaxis in a murine model of food allergy. Allergy.

[11] Cuello-Garcia CA (2015). Probiotics for the prevention of allergy: A systematic review and meta-analysis of randomized controlled trials. J. Allergy Clin. Immunol..

[12] de Moreno de LeBlanc A (2015). Current review of genetically modified lactic acid bacteria for the prevention and treatment of colitis using murine models. Gastroenterol. Res. Pract.

[13] Simpson MR, Dotterud CK, Storrø O, Johnsen R, Øien T (2015). Perinatal probiotic supplementation in the prevention of allergy related disease: 6 year follow up of a randomised controlled trial. BMC Dermatol..

[14] Heselmans M (2015). Gut flora in health and disease: potential role of probiotics. Curr. Issues Intest. Microbiol.

[15] Marco ML, Pavan S, Kleerebezem M (2006). Towards understanding molecular modes of probiotic action. Curr. Opin. Biotechnol..

[16] Lee J, Seto D, Bielory L (2008). Meta-analysis of clinical trials of probiotics for prevention and treatment of pediatric atopic dermatitis. J. Allergy Clin. Immunol..

[17] Vliagoftis H, Kouranos VD, Betsi GI, Falagas ME (2008). Probiotics for the treatment of allergic rhinitis and asthma: systematic review of randomized controlled trials. Ann. Allergy Asthma Immunol..

[18] Boyle RJ, Bath-Hextall FJ, Leonardi-Bee J, Murrell DF, Tang ML (2009). Probiotics for the treatment of eczema: a systematic review. Clin. Exp. Allergy.

[19] Żukiewicz-Sobczak W, Wróblewska P, Adamczuk P, Silny W (2014). Probiotic lactic acid bacteria and their potential in the prevention and treatment of allergic diseases. Cent. Eur. J. Immunol..

[20] Yazdanbakhsh M, Kremsner PG, van Ree R (2002). Allergy, parasites, and the hygiene hypothesis. Science.

[21] Soroosh P, Doherty TA (2009). Th9 and allergic disease. Immunology.

[22] Louten J, Boniface K, de Waal Malefyt R (2009). Development and function of TH17 cells in health and disease. J. Allergy Clin. Immunol..

[23] Smits HH (2005). Selective probiotic bacteria induce IL-10-producing regulatory T cells *in vitro* by modulating dendritic cell function through dendritic cell-specific intercellular adhesion molecule 3-grabbing nonintegrin. J. Allergy Clin. Immunol..

[24] Inoue Y, Iwabuchi N, Xiao JZ, Yaeshima T, Iwatsuki K (2009). Suppressive effects of *bifidobacterium breve* strain M-16V on T-helper type 2 immune responses in a murine model. Biol. Pharm. Bull..

[25] Kwon HK (2010). Generation of regulatory dendritic cells and CD4^+^Foxp3^+^ T cells by probiotics administration suppresses immune disorders. Proc. Natl. Acad. Sci. USA.

[26] Albert MH (2014). MiRNome and transcriptome aided pathway analysis in human regulatory T cells. Genes Immun..

[27] Powell JD, Pollizzi KN, Heikamp EB, Horton MR (2012). Regulation of immune responses by mTOR. Annu. Rev. Immunol..

[28] Wells JM, Mercenier A (2008). Mucosal delivery of therapeutic and prophylactic molecules using lactic acid bacteria. Nat. Rev. Microbiol..

[29] Wells JM, Rossi O, Meijerink M, van Baarlen P (2011). Epithelial crosstalk at the microbiota-mucosal interface. Proc. Natl. Acad. Sci. USA.

[30] Kurashima Y, Goto Y, Kiyono H (2013). Mucosal innate immune cells regulate both gut homeostasis and intestinal inflammation. Eur. J. Immunol..

[31] Carlier FM, Sibille Y, Pilette C (2016). The epithelial barrier and immunoglobulin A system in allergy. Clin. Exp. Allergy.

[32] Lyons A (2010). Bacterial strain-specific induction of Foxp3+ T regulatory cells is protective in murine allergy models. Clin. Exp. Allergy.

[33] Noval RM, Chatila TA (2016). Regulatory T cells in allergic diseases. J. Allergy Clin. Immunol..

[34] Chaudhry A (2009). CD4^+^ regulatory T cells control TH17 responses in a Stat3-dependent manner. Science.

[35] Wakashin H (2008). IL-23 and Th17 cells enhance Th2 cell-mediated eosinophilic airway inflammation in mice. Am. J. Respir. Crit. Care Med..

[36] Jan RL (2012). *Lactobacillus gasseri* suppresses Th17 pro-inflammatory response and attenuates allergen-induced airway inflammation in a mouse model of allergic asthma. Br. J. Nutr..

[37] Zeiser R (2008). Differential impact of mammalian target of rapamycin inhibition on CD4+ CD25+ Foxp3+ regulatory T cells compared with conventional CD4+ T cells. Blood.

[38] Capobianco F (2008). Oral sensitisation with shrimp tropomyosin induces in mice allergen-specific IgE, T cell response and systemic anaphylactic reactions. Int. Immunol..

[39] Li XM (2000). A murine model of peanut anaphylaxis: T- and B-cell responses to a major peanut allergen mimic human responses. J. Allergy Clin. Immunol..

