# Supplementary material for: Lactic acid bacteria-specific induction of CD4+Foxp3+T cells ameliorates shrimp tropomyosin-induced allergic response in mice via suppression of mTOR signaling
Source: Sci Rep. 2017 May 16;7:1987. doi: 10.1038/s41598-017-02260-8 (PMC5434066; doi:10.1038/s41598-017-02260-8)
Supplement: Supplementary file 1 — Lactic acid bacteria-specific induction of CD4+Foxp3+T cells ameliorates shrimp tropomyosin-induced allergic response in mice via suppression of mTOR signaling [file 41598_2017_2260_MOESM1_ESM.pdf]

Supplementary Information

Title: Lactic acid bacteria-specific induction of $CD4^+Foxp3^+T$ cells ameliorates shrimp tropomyosin-induced allergic response in mice via suppression of mTOR signaling

Running head: LAB suppresses ST-induced allergy by regulation of Tregs and mTOR signaling

Linglin Fu[1, 2], Jixiang Peng[1], Shushu Zhao[1], Yan Zhang[3], Xiurong Su[4], Yanbo Wang[1, 2]*

[1]Food Safety Key Laboratory of Zhejiang Province, School of Food Science and Biotechnology, Zhejiang Gongshang University, Hangzhou, 310018, P.R. China

[2]Laboratory of Mucosal Immunology and Food Research, School of Food Science and Biotechnology, Zhejiang Gongshang University, Hangzhou, 310018, P.R. China

[3]Hebei Food Inspection and Research Institute, Shijiazhuang, 050091, P. R. China

[4]School of Marine Sciences, Ningbo University, Ningbo, 315211, P.R. China

Corresponding author: Dr. Y. B. Wang

Address: School of Food Science and Biotechnology, Zhejiang Gongshang University, 18 Xuezheng Road, Xiasha University Town, Hangzhou, 310018, P.R. China

Email: wangyb@mail.zjgsu.edu.cn

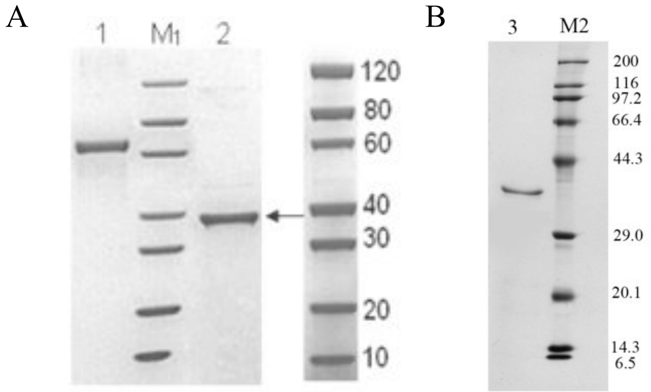

Supplementary Figure S1 SDS-PAGE (A) and Western blot (B) analysis of purified shrimp tropomyosin (ST). Lane 1, BSA (2 μg); lane 2, purified ST (2 μg); lane 3, Western blotted with purified ST; M1 and M2, molecular markers (kDa).

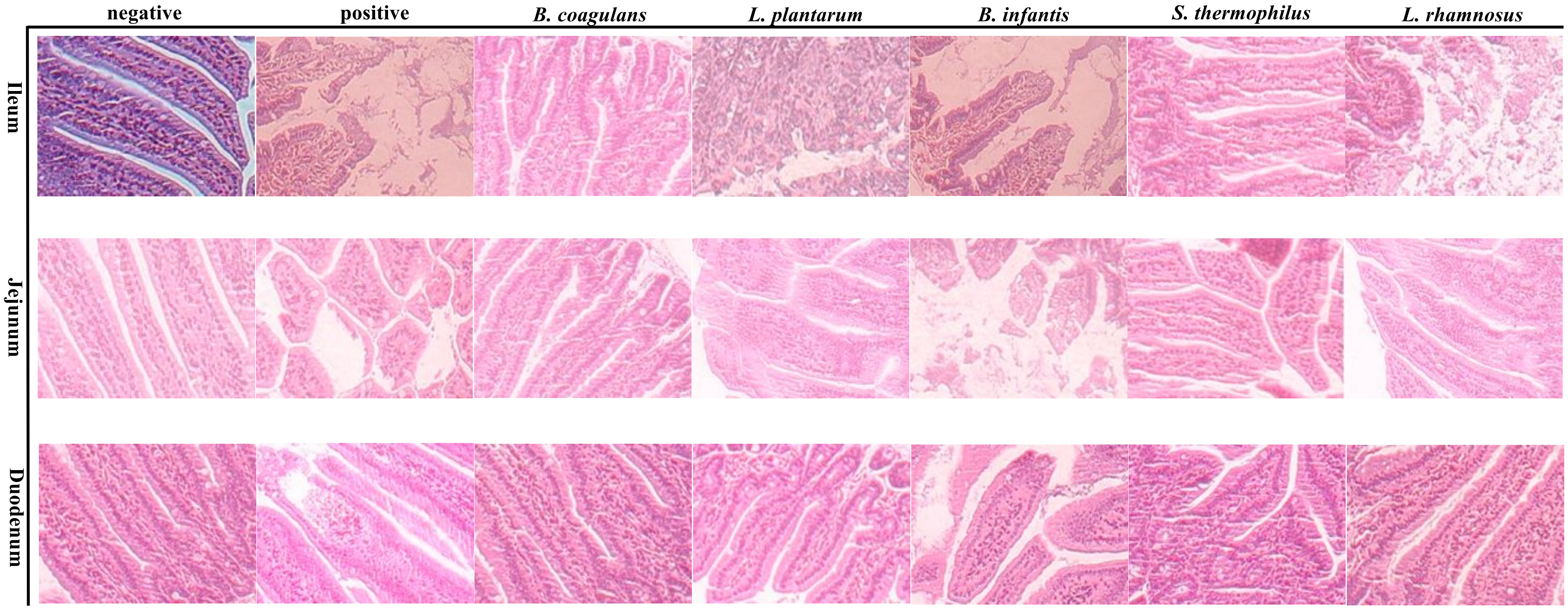

Supplementary Figure S2 Representative intestinal specimens stained with haematoxylin and eosin (HE) after administration of different LAB strains response to ST sensitization. Sections were photographed at × 400 magnifications using a light microscope. The results shown are representative of more than three experiments with similar results.

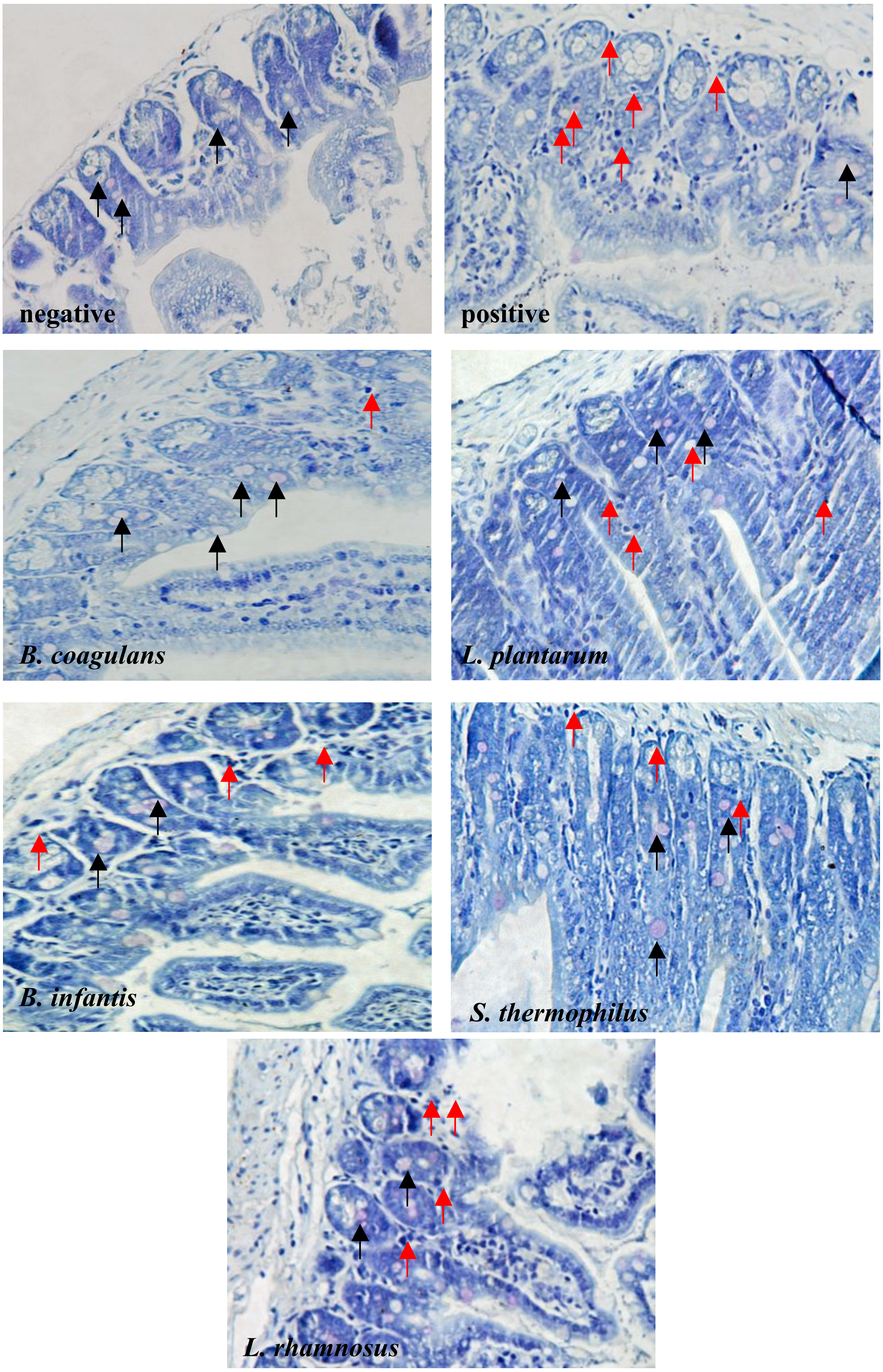

Supplementary Figure S3 Representative intestinal specimens stained with toluidine blue (TB) for degranulated MCs detection in different LAB strain treatments response to ST sensitization. Sections were photographed at × 400 magnifications using a light microscope. The results shown are representative of more than three experiments with similar results. Intact and degranulated MCs are indicated by black and red arrows, respectively.

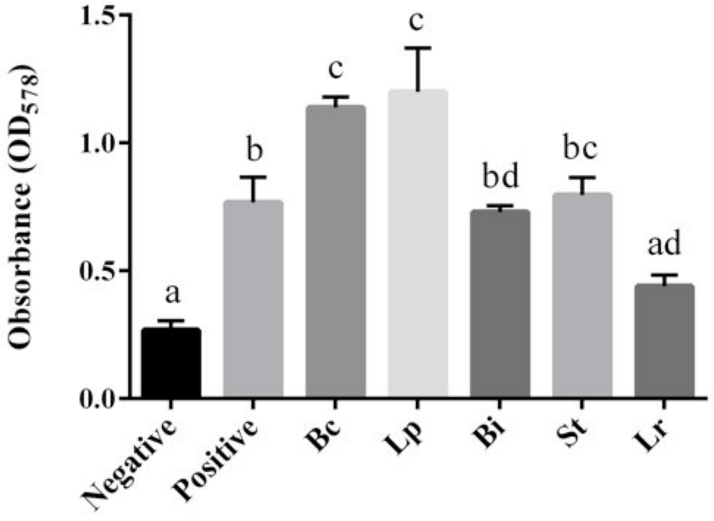

Supplementary Figure S4 Probiotic LAB strains induce proliferation of spleen cells. Proliferation of lymphocytes from the control or probiotic group mice was measured after stimulation with ST. Data are representative of three independent experiments. Results are expressed as means ±SD. Statistical significance ($p < 0.05$) was marked by different letters.

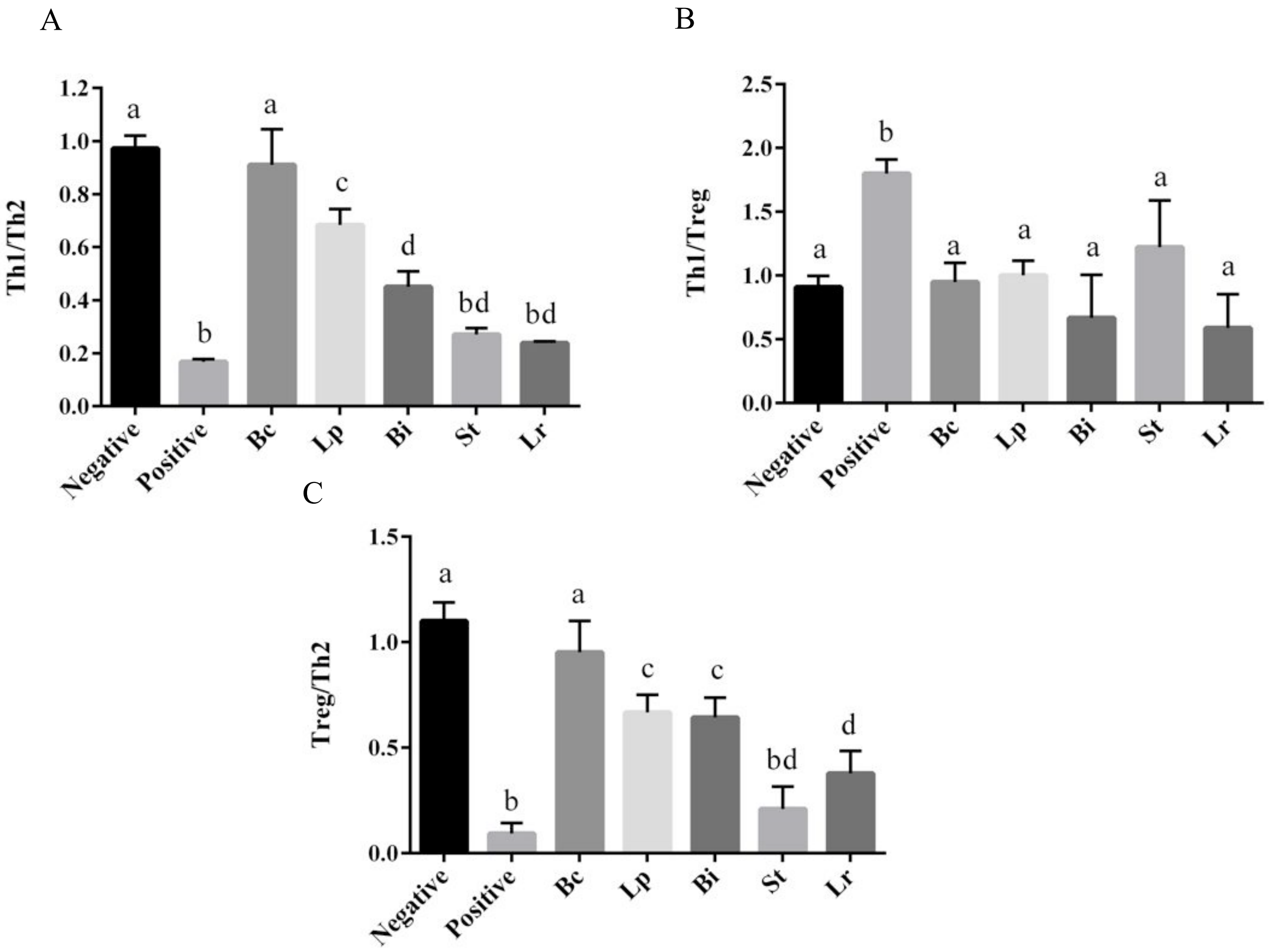

Supplementary Figure S5 The ratios of T cell subsets in the control or probiotic-treated mice analyzed by FACS. The levels of Th1/Th2 (A), Th1/Treg (B), and Treg/Th2 (C) are shown as mean ±SD. Data are representative of three independent experiments. Statistical significance ($p < 0.05$) was marked by different letters.

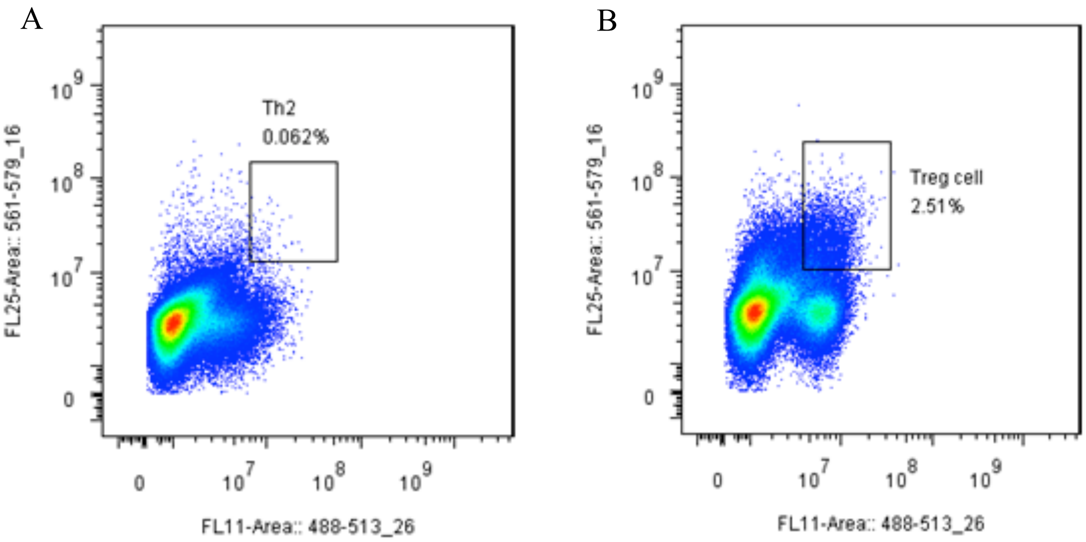

Supplementary Figure S6 Cell sorting by MoFLo Astrios EQ (Beckman Coulter, Brea, CA, USA) flow cytometer. The Th2 (A) and Treg (B) were sorted using monoclonal antibodies of FITC-labeled anti-mouse CD69, PE-labeled anti-mouse ST2 (IL-33R), FITC-labeled anti-mouse CD4 and PE-labeled anti-mouse CD25.

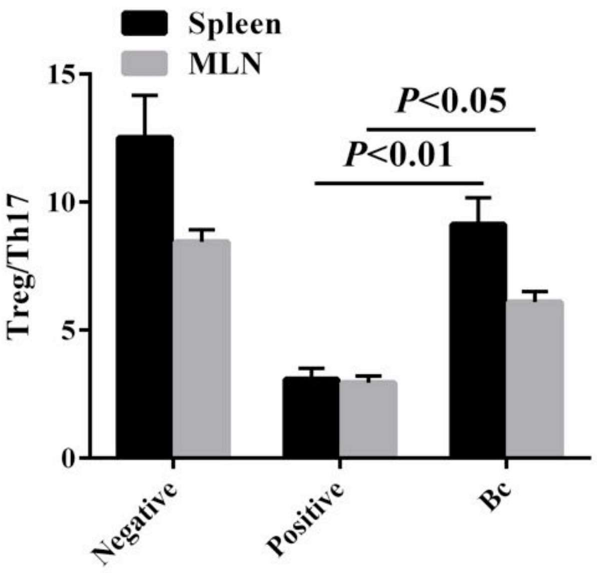

Supplementary Figure S7 The ratios of Treg and Th17 cell subsets in spleen and MLN of the controls or Bc-treated mice analyzed by FACS. The levels of Treg/Th17 are shown as mean ±SD. Data are representative of three independent experiments.

Materials and Methods

Immunohistochemistry

The intestinal samples from positive, negative and Bc groups were fixed in 4% phosphate-buffered paraformaldehyde (pH 7.2), and 5-μm paraffin sections were used for immunohistochemistry staining of lamina propria following the method described previously (*Sun C, Fu LL, Wang Y. Food Agric Immunol 2015; 26:577-89.*). The primary (1:100 dilution of rabbit polyclonal anti-Stat3$^{phospho\ Tyr705}$ and anti-GATA3) and biotinylated secondary (1:400 dilution of goat anti-rabbit IgG, SouthernBiotech) antibodies were added in an appropriate time point, respectively.

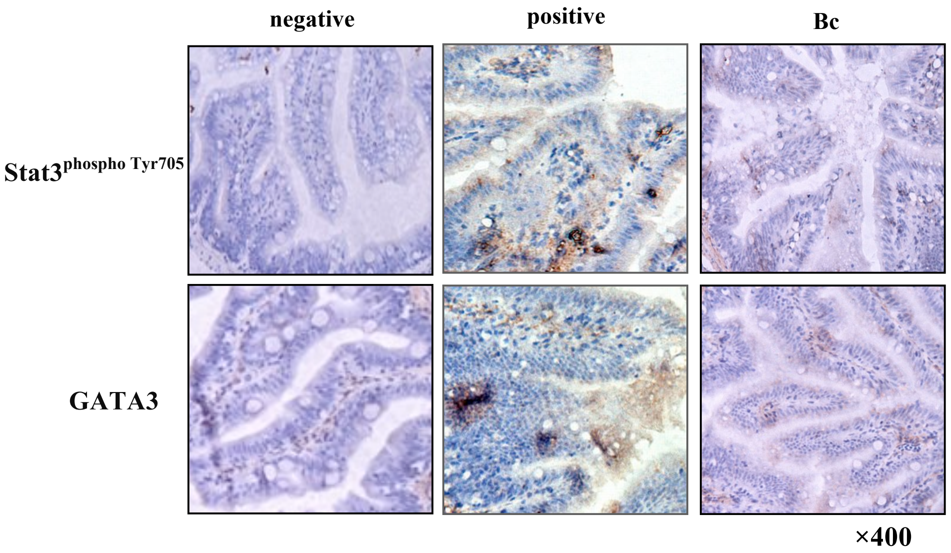

Supplementary Figure S8 Immunohistochemical detection of STAT3$^{phospho\ Tyr705}$ and GATA-3 in lamina propria from the controls or Bc-treated mice upon ST sensitization. Sections were photographed at × 400 magnifications using a light microscope.

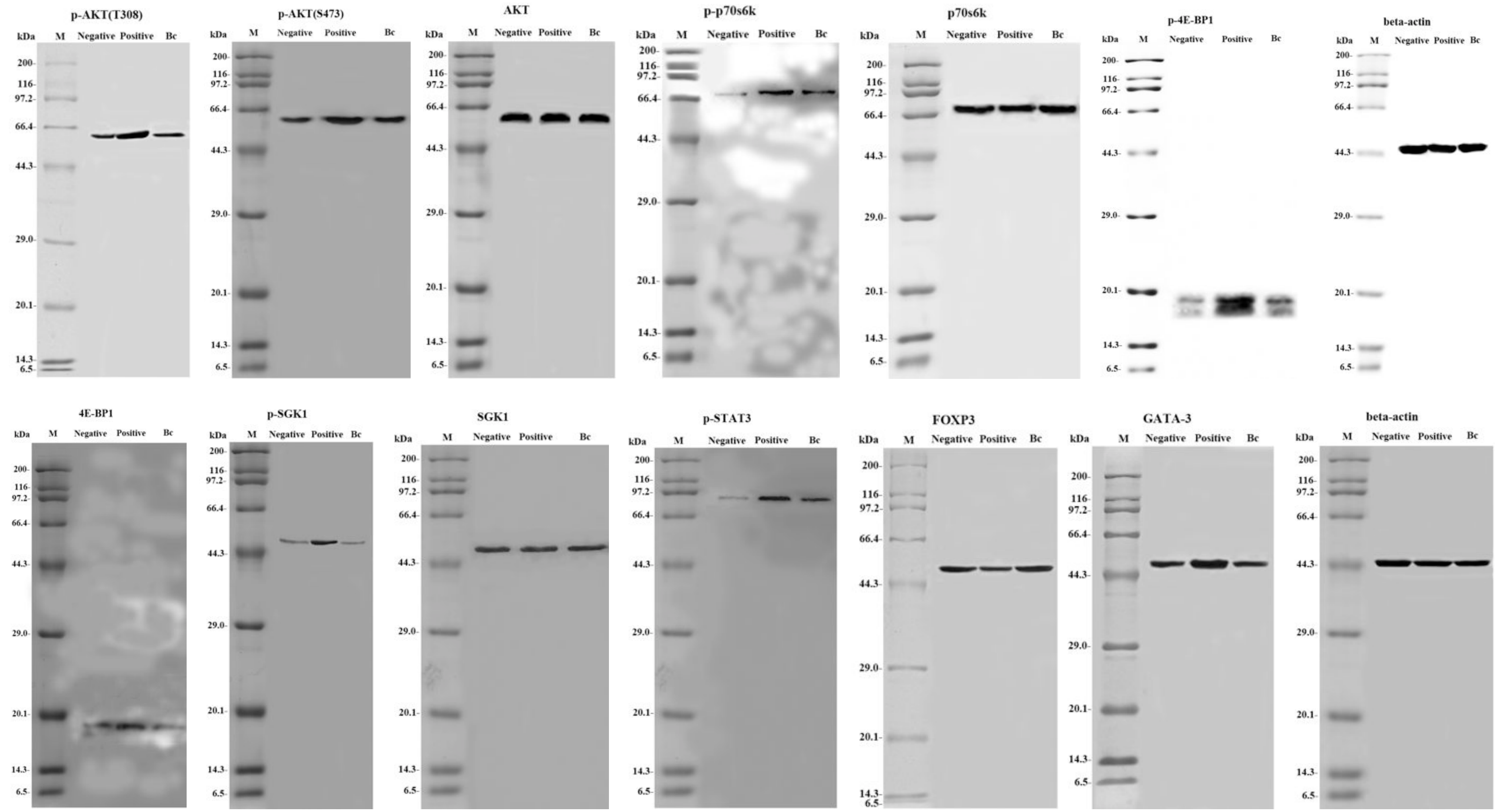

Supplementary Figure S9 Full-length blots. Splenocytes were lysed and immunoblotted with antibodies to SGK1$^{\text{phospho Ser422}}$, pan-Akt$^{\text{phospho T305/308/309}}$, p70S6K$^{\text{phospho Thr389}}$, 4E-BP1$^{\text{phospho Thr37/46}}$, Akt$^{\text{phospho Ser473}}$, Stat3$^{\text{phospho Tyr705}}$, Foxp3, p70S6K, 4E-BP1, Akt, SGK1, GATA3 and **β**-actin. M, molecular marker.
